# A fundamental relationship between TCR diversity, repertoire size and systemic clonal expansion: insights from 30,000 TCR*β* repertoires

**DOI:** 10.3389/fimmu.2025.1707727

**Published:** 2026-01-08

**Authors:** H. Jabran Zahid, Damon May, Harlan Robins, Julia Greissl

**Affiliations:** 1 Microsoft Research, Redmond, WA, United States; 2 Adaptive Biotechnologies, Seattle, WA, United States

**Keywords:** intrinsic clonalty, T cell receptor diversity, immune repertoires, systems immunology, immune homeostasis

## Abstract

**Introduction:**

T cell receptor (TCR) diversity is essential for immune defense, yet the mechanisms underlying its decline with age and its variation among individuals remain poorly understood. These patterns are typically attributed to passive processes such as thymic atrophy and cumulative immune exposures. However, this view does not account for the systematic and highly structured variation in TCR diversity observed across large populations.

**Methods:**

We analyze TCRβ repertoires from approximately 30,000 adults using high throughput sequencing. We quantify repertoire size and the contribution of the most expanded clones and evaluate their ability to predict TCRβ diversity across age, sex and Cytomegalovirus exposure using machine learning and linear modeling approaches.

**Results:**

We show that TCRβ diversity is almost entirely determined by two measurable repertoire features: repertoire size and the frequency of the 1,000 most abundant clones. Together, these features explain 96% of the variance in TCRβ diversity, capture its dependence on age and sex and define a robust relationship that persists under strong immune perturbations such as Cytomegalovirus infection. This relationship arises because the frequency of abundant clones, which represent less than one percent of TCRβ diversity, tracks a repertoire wide pattern of coordinated clonal expansion which we term intrinsic clonality.

**Discussion:**

We propose that intrinsic clonality reflects a fundamental, previously unrecognized property of the immune system which challenges the view that TCR diversity declines primarily through passive erosion. Rather, TCR diversity emerges as a system level property mediated by repertoire size and intrinsic clonality, both of which are likely subject to homeostatic regulation. These findings offer a new conceptual framework for understanding TCR diversity within immune homeostasis which may guide therapies aimed at restoring immune function.

## Introduction

The theory of clonal selection is a cornerstone of modern immunology, providing the foundation for understanding how TCR diversity is broadly shaped and maintained by the immune system (1). T cells play an essential role in immune defense by targeting antigens from infections and cancer, with their specificity determined by T cell receptors (TCRs) (2, 3). The ability to respond to a broad range of pathogens is enabled by the diversity of the TCR repertoire (4–13). A large pool of naive T cells with diverse, randomly rearranged TCRs is primarily generated during childhood and adolescence via V(D)J recombination and is maintained throughout adulthood predominantly through homeostatic proliferation (14, 15). Mechanisms of immune tolerance eliminate or regulate self-reactive T cells, thereby limiting responses to non-self antigens (16). The T cell repertoire is further shaped by selection processes, including clonal expansion upon antigen encounter and the subsequent preferential retention of activated T cells in the memory compartment. A key feature of immune homeostasis is the long-term balance between naive and memory T cell compartments, which enables rapid responses to previously encountered antigens while preserving the capacity to recognize new ones (17, 18).

Despite its essential role, the mechanisms governing TCR diversity remain poorly understood. Diversity declines by about a factor of two between the ages of 20 and 80 years and is systematically lower in males than females (19, 20). Moreover, variation between individuals exceeds that explained by age and sex alone (20). Changes in TCR diversity are commonly attributed to passive, cumulative processes such as thymic atrophy, stochastic cell loss and chronic immune activation (21–29). Under this view, diversity passively erodes over time independent of any intrinsic homeostatic regulatory mechanisms. However, these factors fail to explain the systematic, population-wide patterns observed in large datasets, nor do they identify specific mechanisms that mediate and constrain diversity.

Understanding what determines TCR diversity is critical because it directly impacts the immune system’s ability to recognize novel antigens. Reduced TCR diversity is linked to poor health outcomes including a greater risk of infectious disease and cancer (20, 21, 24, 26, 30– 31). This association with human health highlights the urgent need to understand TCR diversity within the broader context of immune homeostasis. Identifying factors that determine TCR diversity, which may themselves be intrinsically regulated, can provide new mechanistic insight into how TCR diversity is maintained and inform interventions aimed at restoring immune competence after its decline.

Here we analyze TCR*β* repertoires from ∼30,000 individuals and show that diversity is almost entirely determined by two measurable features of the repertoire: the total number of T cells sequenced (repertoire size) and the frequency of the 1,000 most abundant clones. These two quantities independently correlate with TCR*β* diversity and together account for 96% of its variation across individuals, including its systematic dependence on age and sex, as well as its response to Cytomegalovirus (CMV) exposure. The key finding of our analysis is that the predictive power of the frequency of abundant clones arises from the apparently coordinated nature of clonal expansion across the repertoire. We interpret this coordination as the manifestation of a fundamental, system level property that governs the amplitude of clonal expansion across T cells, which we term *intrinsic clonality*. Intrinsic clonality may be a previously unrecognized feature of the immune system subject to homeostatic control, helping to explain how TCR diversity is mediated within immune homeostasis with potentially important implications for translational research.

## Results

We conduct a cross-sectional analysis of 30,430 TCR*β* repertoires processed under standardized protocols in a CLIA^1^-certified laboratory. 95% of subjects in this cohort are aged 20–74 years (median 50) with 47% males and 53% females. The cohort was sequenced as part of the T-Detect COVID test which was granted Emergency Use Authorization by the Food and Drug Administration^2^ and is the same cohort analyzed by Zahid et al. (20). We measure the total number of T cells sequenced, *S*, and the number of unique clonotypes (i.e., richness), *D*, and refer to these quantities as the repertoire size and TCR diversity, respectively; we interpret them as relative measures of the true underlying size and diversity of the peripheral repertoire. Both quantities follow a log-normal distribution across individuals (all logarithmic values refer to base 10). We further define *S*_1000_ as the total number of T cells derived from the 1,000 most abundant clones and *P*_1000_ as the percentage of the repertoire they comprise (i.e., *P*_1000_ = 100 × *S*_1000_*/S*). To facilitate straightforward interpretation, we use *P*_1000_ when visualizing the data. For all modeling applications, *S*_1000_ is used to avoid covariance between *P*_1000_ and *S*, resulting in more robust and interpretable models.

Repertoire size is influenced by both biological factors and technical variables such as input volume and measurement uncertainty. We account for measurement error in our analysis and note that repertoire size strongly correlates with the T cell fraction (i.e., the proportion of peripheral blood mononuclear cells that are T cells; Spearman *ρ* = 0.84). Analyses using either repertoire size or T cell fraction yield consistent results, indicating that our findings are robust to the chosen metric. This supports the conclusion that the relationships we observe reflect intrinsic immune properties rather than technical artifacts.

### TCR*β* diversity, repertoire size and clonal expansion

**Figure 1:**
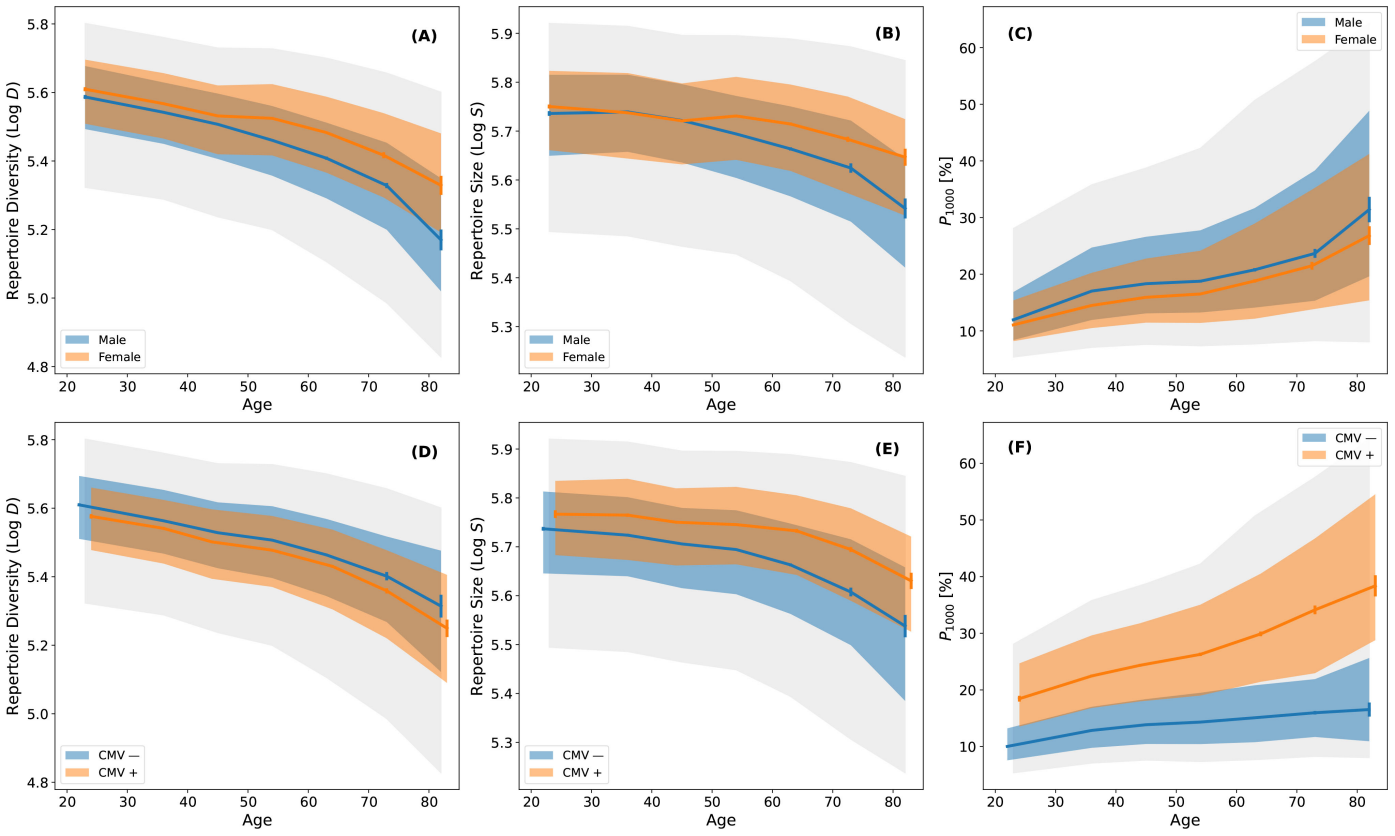
The dependence of T cell receptor diversity, repertoire size and clonal expansion on age, sex and CMV exposure status. **(A)** Log of TCR*β* diversity (Log *D*) as a function of age stratified by sex. Blue and orange curves are the median diversity in decade wide age bins for males and females, respectively. Error bars are bootstrapped and blue and orange shaded regions indicate the distribution of the central 50% of the data. Gray shading represents the central 90% of the subjects. **(B)** Log of the total number of productive TCR*β*s sequenced (Log *S*) in each repertoire as a function of age stratified by sex. The binning procedure and shading definitions are the same as in **(A)**. **(C)***P*_1000_ as a function of age stratified by sex. The binning procedure and shading definitions are the same as in **(A)**. **(D–F)** are the same as in **(A–C)**, respectively, but data are stratified by CMV exposure status rather than sex.

TCR*β* diversity declines with age. This decline occurs ∼10 years earlier in males than in females, leading to pronounced differences emerging in middle age (**Figure 1A**). After accounting for measurement uncertainty, the peak-to-peak intrinsic biological variance in *D* for the central 90% of subjects increases by a factor of 2 to 5 (0.3 dex to 0.7 dex^3^) between the ages of 20 and 80 years, respectively (20). Notably, inter-individual variation in *D* exceeds the systematic effects of age, sex and CMV exposure, particularly among older individuals. Repertoire size (*S*) declines with age similarly to *D* (**Figure 1B**), while clonal expansion (*P*_1000_) increases from 10% to 30% between ages 20 and 80 and is consistently lower in females (**Figure 1C**). Furthermore, CMV-positive individuals exhibit slightly lower TCR*β* diversity, substantially larger repertoire size and higher clonal expansion (**Figures 1D–F**). These findings demonstrate that repertoire size, TCR*β* diversity and clonal expansion all depend on age, sex and CMV exposure status.

**Figure 2:**
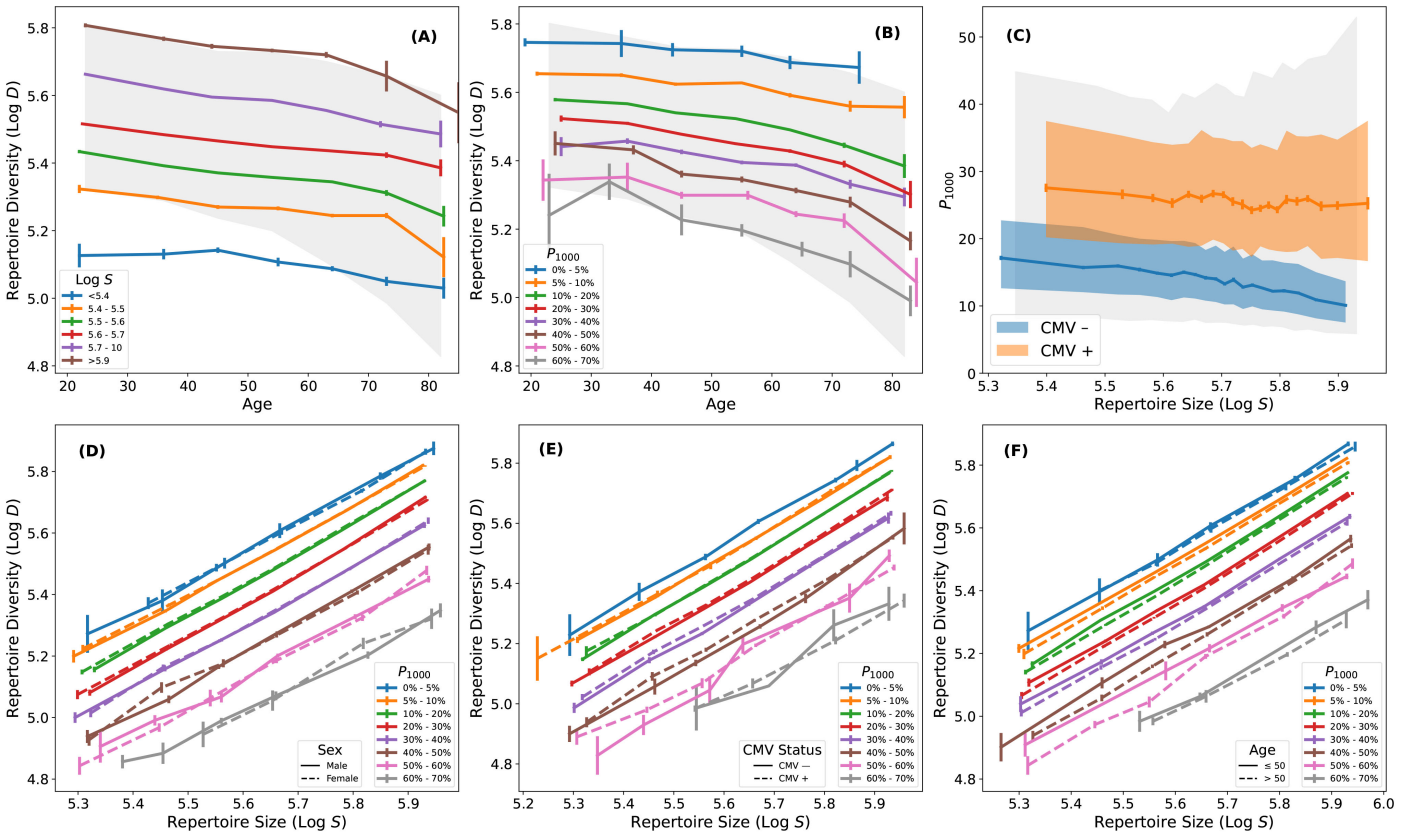
Relationship between T cell receptor diversity, repertoire size and clonal expansion. **(A)** Log *D* as a function of age with colored curves indicating median Log *D* in bins of Log *S*. Gray shading represents the central 90% of subjects. TCR*β* diversity increases with repertoire size. **(B)** Same as in **(A)** but with color bars indicating median Log *D* as function of age in bins of *P*_1000_. TCR*β* diversity decreases with increasing clonal expansion. **(C)** Relationship between repertoire size and *P*_1000_ stratified by CMV exposure status. For CMV-positive subjects, there is a marginal anti-correlation between *P*_1000_ and *S* (Spearman *ρ* = −0.04, *p* = 7×10^−5^). For CMV-negative subjects, the anti-correlation is significantly stronger (Spearman *ρ* = −0.25, *p* = 1×10^−264^). **(D)** Log *D* as a function of Log *S* with colored curves indicating median Log *D* in bins of *P*_1000_. The solid and dashed curves are for males and females, respectively. **(E)** Same as **(D)** but solid and dashed curves are for CMV-negative and CMV-positive subjects, respectively. **(F)** Same as in **(D)** but solid and dashed curves are subjects that are younger and older than the median age of the cohort (50 years), respectively. After accounting for repertoire size and clonal expansion, TCR*β* diversity is independent of sex, CMV exposure status and age.

TCR*β* diversity is independently correlated with both repertoire size and clonal expansion. We calculate the median *D* as a function of age, binned by *S* and *P*_1000_, respectively (**Figures 2A, B**). At all ages, individuals with high *D* tend to have low *P*_1000_ and high *S*, and vice versa. In CMV-negative individuals, larger repertoire size is accompanied by a more balanced clonal distribution, with both factors contributing to higher TCR*β* diversity (**Figure 2C**). Conversely, in CMV-positive subjects, repertoire size and clonal expansion are decoupled, such that large repertoires coexist with high levels of clonal expansion. Larger repertoire sizes compensate for increased clonality in CMV-positive individuals, thereby mitigating the impact of CMV exposure on TCR*β* diversity (32).

At a fixed *S*, *D* systematically declines with increasing *P*_1000_, demonstrating that clonal expansion reduces diversity independent of total repertoire size (**Figures 2D–F**). Remarkably, after controlling for *S* and *P*_1000_, the residual dependence of *D* on age, sex or CMV exposure status is minimal, indicating that these biological factors influence TCR diversity through their effects on repertoire size and clonal expansion. Meaning, repertoire size and clonal expansion strongly mediate the observed dependence of TCR diversity on age, sex and CMV exposure, suggesting that the relationship between these repertoire measures is largely independent of age, sex and CMV exposure.

Variations in repertoire size and clonal expansion almost fully account for the observed variance in TCR*β* diversity at any given age (**Figures 2A, B**). *P*_1000_ increases systematically with age (**Figure 1C**) but subjects with low *P*_1000_ and high *D* are present at all ages. Notably, the 1000 most abundant clones may occupy a sizable fraction of the repertoire but they represent a very small fraction of the diversity (*<* 1%). Moreover, the 1000 most abundant clones in any repertoire are dominated by CD8^+^ memory T cells (**Supplementary Figure S1**), but we find that TCR*β* diversity (*D*) is strongly correlated with the average clonal expansion of all clones in the repertoire (Spearman *ρ* = 0.92), which includes CD4^+^ and naive T cells. This correlation remains significant even when the 1000 most abundant clones are excluded (Spearman *ρ* = 0.52), indicating that *P*_1000_ serves as a proxy for the repertoire wide property we refer to as intrinsic clonality.

### Modeling TCR*β* diversity

To better understand the factors shaping TCR diversity, we develop predictive models using biological and repertoire-derived features. We first use XGBoost, a gradient-boosted decision tree algorithm (33), to model TCR*β* diversity as a function of age, sex and CMV exposure status.

**Figure 3:**
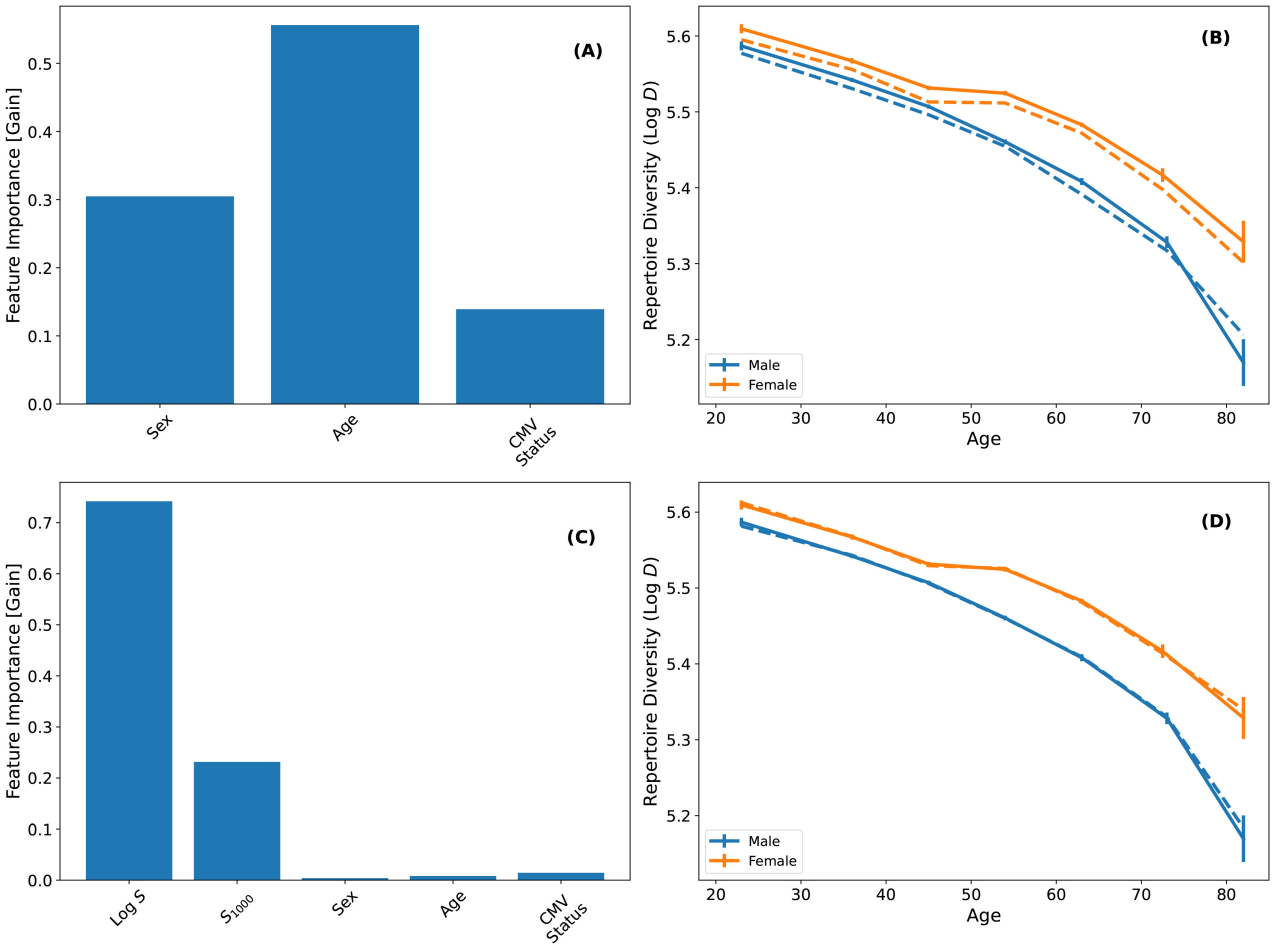
Modeling TCR*β* diversity using XGBoost. **(A)** Feature importance of sex, age and CMV status in predicting TCR*β* diversity. **(B)** Solid lines show the median Log *D* as a function age stratified by sex. The dashed lines show the model predictions generated via five-fold crossvalidation. **(C)** Feature importance when including Log *S* and *S*_1000_ as features in the model. The negligible contribution of age, sex and CMV status demonstrates that repertoire size and *S*_1000_ account for the dependence of TCR*β* diversity on these factors. **(D)** Same as **(B)** but for a model including repertoire size and *S*_1000_ as parameters. Repertoire size and *S*_1000_ robustly predict TCR*β* diversity and are better predictors of its systematic dependence on age and sex than the model shown in **(A, B)**.

All three features are predictive (**Figure 3A**) and the model broadly captures the systematic dependence of TCR*β* diversity on these variables (**Figure 3B**). Next, we include repertoire size (*S*) and clonal expansion (*S*_1000_). Adding these features substantially improves model performance (**Figures 3C, D**): *S* and *S*_1000_ together explain approximately 96% of the intrinsic variance in TCR*β* diversity (see Materials & Methods). Furthermore, including *S* and *S*_1000_ eliminates the predictive value of sex, age and CMV status, confirming that their effects on TCR diversity are mediated through repertoire size and intrinsic clonality.

The relationship between TCR*β* diversity, repertoire size and intrinsic clonality is independent of age, sex and CMV exposure status. We fit a linear model to explicitly quantify this relationship:

(1) $$\hat{D}\left(S,S_{1000}\right)=\left(0.825\pm 0.001\right)\cdot S-\left(0.965\pm 0.003\right)\cdot S_{1000}.$$

**Figure 4:**
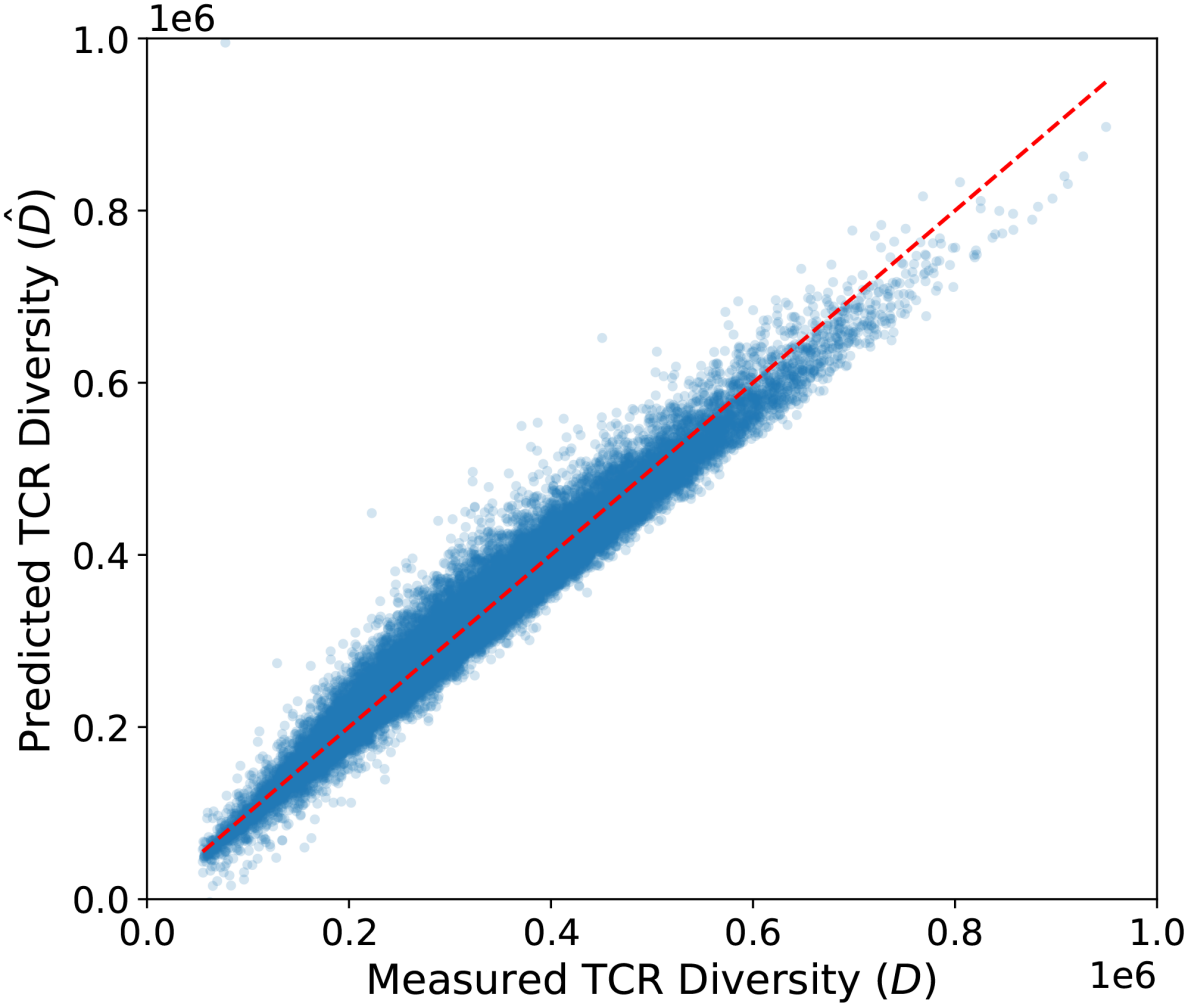
A linear model of TCR*β* diversity. We model *D* as a linear function of *S* and *S*_1000_ and fit the data in five-fold cross-validation. We plot the predicted TCR*β* diversity as a function the measured TCR*β* diversity. The red dashed line shows one-to-one correspondence. The linear model accurately describes the measured TCR*β* diversity (*R*^2^ = 0.96).

Here $\;\hat{D}$ represents the predicted TCR*β* diversity as a linear function of *S* and *S*_1000_. We note that all variables in the model are expressed in their original, non–log-transformed values. Equation 1 describes *D* as increasing with *S* and decreasing with *S*_1000_. Notably, the coefficient of *S*_1000_ is close to unity, indicating that TCR*β* diversity decreases nearly one-to-one with increasing *S*_1000_. The model yields an *R*^2^ of 0.96 (**Figure 4**), consistent with the ∼4% residual intrinsic scatter estimated from our XGBoost model, further supporting the robustness and completeness of the relationship. These results reinforce the idea that *S*_1000_ and *P*_1000_ serve as quantitative proxies for intrinsic clonality, a systemic repertoire property that mediates TCR diversity. Despite CMV-positive and CMV-negative individuals exhibiting distinct relationships between repertoire size and intrinsic clonality (**Figure 2C**), both groups follow a consistent relationship linking these variables to TCR diversity, underscoring the universal and fundamental role of this relationship in characterizing immune homeostasis.

We note that the choice of the 1,000 most abundant clones is not inherently special. For instance, when using the 10 or 100 most abundant clones (*S*_10_ or *S*_100_, respectively) as features (**Supplementary Figure S3**), the model performance is only slightly degraded—likely due to greater statistical uncertainty in these measures. The fitted coefficients differ modestly from those obtained using *S*_1000_, reflecting the smaller dynamic range of these quantities, but the overall linear relationship remains intact. Importantly, even with *S*_10_ or *S*_100_, the model continues to account for the dependence of TCR*β* diversity on age and sex, demonstrating that the form of the relationship is robust to the precise definition of our proxy for intrinsic clonality. This result reinforces that the relationship reflects the heavy-tailed structure of the repertoire rather than a privileged cutoff. Additionally, we find that low TCR*β* diversity does not appear to be linked to any specific or specialized set of immune exposures and HLA genotypes are not predictive (see **Supplementary Material**). Taken together, these results strongly suggest that repertoire size and intrinsic clonality are the primary determinants of TCR diversity.

## Discussion

TCR diversity is essential for immune competence, enabling the recognition and elimination of diverse threats. We show that repertoire size (*S*) and the abundance of the most expanded clones (*S*_1000_) explain nearly all the variation in TCR*β* diversity (*D*) across individuals, including its systematic dependence on age, sex and CMV exposure. We emphasize that this relationship is not a mathematical artifact or tautology. Although *S*_1000_ is derived from a subset of highly expanded clones that represent <1% of TCR*β* diversity, it robustly predicts *D*, a global property of the repertoire. This unexpected result indicates that a small fraction of clones encodes information about the entire distribution. We interpret this coordinated pattern of clonal expansion as the measurable manifestation of a previously unrecognized property of the immune system we term intrinsic clonality. Our proxy of intrinsic clonality does not depend on selecting the 1,000 most abundant clones. Using as few as 10 or 100 most abundant clones yields consistent results, indicating that intrinsic clonality captures a key biological property of the immune system. Thus, our findings reveal a simple but powerful organizing principle: overall TCR diversity is an emergent property of the immune system, arising from a fundamental relationship between repertoire size, intrinsic clonality and TCR diversity itself.

CMV exposure underscores the generality and resilience of the relationship between *D*, *S* and *S*_1000_, demonstrating that it holds even under strong immune perturbations. Unlike acute infections such as SARS-CoV-2 or other chronic herpes viruses like Epstein-Barr Virus, which have significantly smaller and more transient effects on the repertoire, CMV strongly perturbs homeostasis by increasing both repertoire size and intrinsic clonality. The chronic nature of CMV alone does not fully explain its outsized impact on repertoire structure and the biological reasons for its influence remain incompletely understood (34). Nevertheless, the consistency of the relationship between *D*, *S*, and *S*_1000_ across CMV-exposed and -unexposed individuals underscores its fundamental and robust nature.

We find no evidence that shared immune exposures or unmodeled host factors explain the relationship between *D*, *S*, and *S*_1000_. HLA genotype does not predict *D* and both the most abundant clones and their co-occurrence patterns vary widely across individuals, consistent with their origin from disparate immune exposures (**Supplementary Figure S4**). Additionally, a targeted search for TCR*β*s associated with low-diversity repertoires identified no strong candidates, further suggesting that unrecognized shared exposures are not the primary driver of diversity loss (see **Supplementary Methods**). However, these findings are not central to our conclusions. Rather, the key result is that *S* and *S*_1000_ together explain 96% of the variation in TCR*β* diversity, leaving little room for additional contributors. While other variables may correlate with these quantities, repertoire size and clonality are fundamental properties of the T cell repertoire. The predictive power of *S*_1000_ reflects a coordinated pattern of clonal expansion across compartments and is robust to the specific number of clones included in the calculation (**Supplementary Figure S3**). Notably, *S*_1000_ is dominated by memory CD8^+^ T cells (**Supplementary Figure S1**), while *D* is shaped primarily by naive CD4^+^ T cells (35). The near one-to-one inverse relationship between *S*_1000_ and *D* (Equation 1) supports systemic coordination, possibly explaining the observed stability of clonal hierarchy over time (36). Together, these findings point to intrinsic, homeostatic regulation of the T cell repertoire rather than extrinsic factors.

Although the precise mechanisms remain uncertain, we propose that an immunometabolic regulatory network comprising two interdependent homeostatic processes may plausibly underlie our observations. The first regulates repertoire size through T cell competition for soluble IL-7 and IL-15 and access to stromal niches, which together determine how many total T cells can be sustained (17, 37–40), effectively setting a molecular homeostatic point for T cell carrying capacity. The second governs the overall amplitude of clonal expansion through an integrated network of cytokine, metabolic and costimulatory signals that integrate through the mTOR pathway (41, 42). mTOR links the cytokine milieu to cellular metabolic state, effectively coordinating the strength of clonal expansion across the repertoire, giving rise to the system-wide property we term intrinsic clonality. These two homeostatic processes are coupled through cytokine availability, which naturally accounts for the negative correlation between repertoire size and intrinsic clonality observed in CMV-negative subjects. Increased mTOR activity promotes greater clonal expansion but also likely enhances metabolic and cytokine dependence of each cell (43, 44), intensifying competition for limited resources and resulting in a smaller overall repertoire. In contrast, the same constraints do not apply to CMV-specific T cells which are dominated by late-differentiated CD45RA^+^ CCR7^−^ (TEMRA) cells. TEMRAs are largely maintained through IL-15 trans-presentation and do not rely on the soluble cytokine–mediated homeostasis that governs the rest of the repertoire (34, 45). This lack of dependence on cytokine-mediated feedback explains why in CMV-positive individuals the expansion of CMV-specific T cells is balanced by an increase in repertoire size that preserves TCR diversity and why repertoire size and intrinsic clonality become uncoupled. While our findings robustly identify repertoire size and intrinsic clonality as key regulatory parameters, the specific biological mechanisms underlying intrinsic clonality remain speculative and may involve multiple, potentially overlapping pathways beyond mTOR.

There is growing evidence that many aspects of immune repertoire organization are genetically controlled, consistent with heritable regulation of the cytokine and metabolic pathways that govern T cell homeostasis. Cytokine levels and T cell counts vary systematically with age, sex and genetic background (46–53), indicating genetically encoded set points that shape T cell homeostasis. Genetic variation beyond HLA influences cytokine signaling and broader immune traits (47, 54–57), and twin studies demonstrate heritability in responses to homeostatic cytokines such as IL-7 and IL-2 (58) as well as in global immune parameters (46). Variation in genes encoding components and regulators of the mTOR pathway also modulates pathway activity and immune function, indicating that the metabolic arm of this regulatory network is likewise under genetic control (47, 58, 59). Together, these findings suggest that the mechanisms of immune homeostasis are at least partly genetically encoded, while environmental factors likely act through these intrinsic pathways (60–63).

Our analysis captures population-level trends in a cross-sectional manner. Small studies suggest TCR diversity is stable over short periods but declines with age (64, 65). Because our study focuses on adults, it primarily captures homeostatic regulation of established repertoires. In children, thymic production and developmental selection dominate repertoire dynamics. Applying this framework to pediatric cohorts, such as those described by Mitchell et al. (66), could help identify how homeostasis emerges. While these studies are consistent with our finding, their small sizes underscore the need for large-scale, longitudinal studies to help establish intrinsic clonality as a repertoire-wide feature. Investigating links between our findings and immunosenescence and inflammaging (67, 68) may offer further insights, as the mechanisms we propose may help explain key aspects of these age-associated phenomena. Future work could assess whether interindividual variation in mTOR pathway activity predicts intrinsic clonality and repertoire organization at the population level. These efforts could be integrated with studies leveraging high-throughput proteomics in large cohorts to elucidate how systemic cytokine levels influence TCR diversity and to identify molecular mediators of immune homeostasis. Continued integration of immune repertoire data with genomic profiling may help clarify how genetic variation modulates repertoire structure through the regulatory mechanisms we describe. Our findings provide a conceptual framework for investigating how intrinsic and extrinsic forces jointly regulate immune homeostasis, with implications for aging, disease susceptibility and therapeutic intervention.

T cells are essential for maintaining human health and their dysregulation contributes to a wide range of diseases, motivating therapeutic efforts to restore immune function (69, 70). A consistent feature of immune dysfunction is the loss of TCR diversity which is linked to poor clinical outcomes (20, 21, 24, 26, 30, 31, 71). Our findings suggest that TCR diversity is not directly regulated but instead emerges from clonal dynamics governed by repertoire size and intrinsic clonality, two properties that are likely directly regulated within immune homeostasis. By identifying these core determinants, our work provides guidance for therapeutic efforts aimed at preserving TCR diversity and restoring immune balance.

## Materials and methods

### Sequencing of human samples

Details of the sequencing data and IRB information are provided in Zahid et al. (20), here we highlight the most salient information. The CDR3 of TCR*β* chains of T cells is sequenced with a multi-plexed PCR typically using 18*µ*g of genomic DNA (72–75). The median sequencing depth is 518,618 TCR*β*s with 95% of subjects having a sequencing depth between 222,082 and 853,647. The median TCR*β* diversity is 319,802 and 95% of subjects have values between 120,576 and 597,890. 95% of subjects have ages ranging between 20 and 74 years with a median age of 50 years. Sex is self-identified with males comprising 47.2% and females comprising 52.5% of subjects.

### T cell based CMV diagnostic

We use a sensitive and specific T cell based diagnostic on the T Detect Covid cohort to identify subjects exposed to CMV. We use a method previously described in (76–78), which statistically identifies disease associated TCR*β*s based on serologically labeled cases and controls. 2181 labeled samples were used to build a CMV classifier with an area under the receiver operating characteristic curve (AUROC) of 0.96, measured on the same holdout set used in Emerson et al. (76). The performance of the T cell based test is comparable to serology and is limited by the accuracy of the serological labels. This diagnostic test allows us to identify subjects who are exposed to CMV using only their sequenced repertoire. Zahid et al. (20) demonstrate that CMV exposure primarily impacts TCR repertoire size.

### Fitting TCR*β* diversity

We first fit TCR*β* diversity using the XGBRegressor routine implemented in version 2.1.6 of the XGBoost algorithm (33). We select XGBoost because of its ability to capture non-linear relationships, its strong out-of-the-box performance and its flexibility handling categorical variables like sex and CMV exposure status. We adopt the default hyperparameters of the algorithm and use its default squared error loss function. We derive predictions of TCR*β* diversity using a five-fold cross-validation scheme implemented in the routine cross val predict from the scikitlearn (79) package version 1.2.0. We fit the model to a random 80% of the data and predict on the remaining 20%. This process is repeated across five distinct, randomly generated 80/20 splits of the data, ensuring that every data point is predicted without being used for model fitting. We determine feature importance by fitting all the data simultaneously.

We next fit the TCR*β* diversity using a linear model described in Equation 1. We generate predictions in five-fold cross-validation and derive parameters by fitting all the data. We optimize the two parameters using the the optimize.curve fit module in version 1.15.2 of the SciPy package (80). We fit a linear model using the full dataset and generated parameter uncertainties using bootstrap resampling. Model evaluation was performed using five-fold crossvalidation and **Figure 4** shows predicted versus observed TCR*β* diversity values under this validation framework. The reported coefficients and uncertainties reflect the best-fit values and 1*σ* bootstrapped error estimates.

### Estimating residual intrinsic scatter

To quantify the residual intrinsic (biological) scatter in the XGBoost model’s prediction of TCR*β* diversity, we estimate and subtract the contribution of measurement error as:

(2) $$\sigma_{i}=\sqrt{\sigma_{r}^{2}-\sigma_{m}^{2}}.$$

Here *σ_i_*is the intrinsic biological scatter, *σ_r_*is the model uncertainty and *σ_m_*is the measurement uncertainty. The rationale is that for a perfect model the residual intrinsic scatter would be *σ_i_*= 0, meaning the model’s uncertainty would be entirely limited by the measurement error.

Given that measurement errors in repertoire size and TCR*β* diversity are correlated, we estimate the minimum achievable error (MAE) based on variability in *D/S*, the ratio of TCR diversity to repertoire size. Using repeat independent measurements from the same subjects, we calculate the MAE as the standard deviation of differences in Log *D/S*, yielding 0.027 dex (see **Supplementary Material**; **Supplementary Figure S2**). We fit TCR*β* diversity using only *S* and *S*_1000_ as features and find the standard deviation of the fit residuals to be 0.031 dex. Subtracting the MAE from model uncertainty in quadrature yields a residual scatter of 0.015 dex, indicating that approximately 4% of the intrinsic variability in TCR*β* diversity remains unexplained by *S* and *S*_1000_.

## Data Availability

Data tables with TCR repertoire metrics available at https://doi.org/10.5281/zenodo.14976210 and https://doi.org/10.5281/zenodo.13993996.

## Footnotes

1 Clinical Laboratory Improvement Amendments of 1988

2
https://www.fda.gov/media/146481/download

## Supplementary material

Supplementary Figure 1 Distribution of clone frequencies for various compartments. **(A)** and **(B)** show clonal frequencies versus clone ranks derived from the sorted repertoires of 45 subjects split by CMV-negative and CMV-positive subjects, respectively. T cells are sorted on memory/naive and CD4/CD8 markers prior to sequencing. Details of the sorting procedure are provided in Zahid et al. (81). The 1000 most abundant clones are dominated by memory CD8^+^ T cells.

Supplementary Figure 2 Measurement uncertainty and XGBoost model residuals. **(A)** Distribution of of the difference in the quantity Log *D/S* determined from 396 repeat samples. To avoid overestimating measurement uncertainties due to a small number of outliers, we estimate the standard deviation of the distribution by fitting a Gaussian. We attribute this measure from repeat samples of the same subjects as an estimate of the measurement uncertainties and adopt the standard deviation as the minimum achievable error for any model that is not overfitting to the data. **(B)** Distribution of residuals of the XGBoost model fit of TCR diversity using *S* and *S*_1000_ as features. The model error is only slightly larger than the measurement uncertainty, indicating that the model accounts for nearly all the intrinsic biological scatter in the data.

Supplementary Figure 3 Modeling TCR diversity with *S*_100_ and *S*_10_ instead of *S*_1000_. **(A)** and **(B)** are the same as **Figures 2A, B**, respectively, but for a model fit using the fraction of repertoire comprised of the 100—not 1000—most abundant clones, i.e., *S*_100_. **(C)** Distribution of residuals of the model fit with *S* and *S*_100_. **(D)**, **(E)** and **(F)** are the same as **(A, B)** and **(C)**, respectively, for a model using *S*_10_ instead of *S*_100_. Model performance is only slightly degraded when using *S*100 or *S*10 instead of *S*1000.

Supplementary Figure 4 Distribution of the number of TCR*β*s in the 1000 most abundant clones that are HLA-associated and their cluster membership. **(A)** Histogram of the number of TCR*β*s that are one of the 1000 most abundant which are also HLA-associated public TCR*β*s for all subjects. **(B)** Histogram of the number of distinct HLA-associated TCR clusters represented by the TCR*β*s in **(A)** for all subjects. Each cluster is interpreted as mapping to a distinct immune exposure.
